# Healthy food choices in Morocco: A survey on knowledge, attitudes, and practices

**DOI:** 10.1016/j.pmedr.2025.103207

**Published:** 2025-08-09

**Authors:** Morad Kaddouri, Omar Ait El Alia, Mohamed Bouhrim, Mohammed Al-zharani, Fahd A. Nasr, Ashraf Ahmed Qurtam, Said Ihbour, Khalid Boutoial

**Affiliations:** a Laboratory of the Engineering and Applied Technologies, Higher School of Technology, Sultan Moulay Slimane University, Beni Mellal 23000, Morocco; b Laboratoires TBC, Laboratory of Pharmacology, Pharmacokinetics, and Clinical Pharmacy, Faculty of Pharmaceutical and Biological Sciences, P.O. Box 83, F-59000 Lille, France; c Biology Department, College of Science, Imam Mohammad Ibn Saud Islamic University (IMSIU), Riyadh 11623, Saudi Arabia; d Research Team in Sports Sciences, Institute of Sports Sciences, Hassan I University, Settat, Morocco

**Keywords:** Healthy diet, Knowledge–attitude–practice approach, Morocco, Healthy food choices, Healthy policies

## Abstract

**Objectives:**

Understanding the knowledge, attitudes, and practices (KAP) that shape adult eating behaviors is essential for improving nutrition outcomes. This study addresses this need by examining these factors among Moroccan adults—an area often overlooked in research that has primarily focused on nutrient and energy intake.

**Methods:**

A cross-sectional online survey was conducted from October 2024 to January 2025 across all the 12 Moroccan regions. A 38-item KAP questionnaire (validated via back-translation, Cronbach's alpha >0.7) was completed by one thousand thirty-five adults. Data were analyzed using descriptive statistics, analysis of variance, and multiple regression.

**Results:**

Participants showed good knowledge (mean score: 4.46/7 ± 1.2), positive attitudes (6.32/8 ± 1.23), and moderate practices (25.83/44 ± 4.69). Women, urban residents, and higher-income groups scored significantly higher (*p* < 0.05). Significant correlations linked knowledge, attitudes, and practices (*β* = 0.32–0.53*, p* < 0.01).

**Conclusions:**

Despite adequate knowledge, practices lagged, particularly in rural and lower-income groups. Tailored nutrition education and policies addressing socioeconomic disparities may be warranted.

## Highlights

• Most Moroccan adults understand the benefits of fruits and vegetables.
• Urban populations showed stronger nutrition knowledge than rural residents.
• Women had better nutrition knowledge; men had more positive dietary attitudes.
• Across regions, higher income was linked to healthier food habits.

## 1 Introduction

Nutrition is a critical component of human health, serving as an essential element for physical development, prevention of diseases, and the promotion of overall well-being (WHO, 2022). A balanced and diversified diet is imperative for maintaining optimal health and reducing the risk of non-communicable diseases (NCDs), these include obesity, cardiovascular diseases, and type II diabetes (Mozaffarian, 2016). As stated by the World Health Organization, nearly 74 % of worldwide deaths are attributed to NCDs, with a rising prevalence in developing countries, including Morocco (WHO, 2021). Among the primary contributors to these conditions are unhealthy dietary practices, marked by excessive consumption of sugar, saturated fats, and processed foods (Fernandez-Alonso et al., 2024).

In Morocco, the nutritional transition has accelerated over the last few decades. Driven by urbanization and lifestyle changes, traditional, nutrient-rich diets have gradually been replaced by a diet dominated by fast food and industrial products (Kaddouri et al., 2025). This evolution has led to a double burden of malnutrition, characterized by the persistence of undernutrition and the increase in overweight and obesity (Bouhoudan et al., 2024). From 2011 to 2018, the prevalence of overweight rose from 50.8 to 53.0 %, while the prevalence of obesity increased from 17.9 to 20.0 %. These proportions are particularly pronounced among the female population, with 63.4 % of women being overweight and 29.0 % obese (Boukrim et al., 2021).

To address this issue, Morocco has implemented a national nutrition strategy with a focus on four pillars: food security, the integration of nutrition into healthcare, capacity building, and monitoring and evaluation (Ministry of health, 2019). The Hind and Benmoussa, 2019 National Nutrition Survey further revealed widespread micronutrient deficiencies in key micronutrients such as iodine, iron, vitamins A and D, thus underscoring the need for targeted nutritional interventions (Ministry of health, 2019).

Several published studies have established significant correlations between Morocco's ongoing nutritional transition and the escalating prevalence of diet-related chronic conditions, particularly obesity and micronutrient deficiencies (Boukrim et al., 2021; Salaheddine et al., 2015; Talha et al., 2024). While existing research has extensively quantified macronutrient intake patterns and dietary behaviors (Aboussaleh et al., 2009; Allali, 2017; Bouhoudan et al., 2024), a critical knowledge gap persists regarding the cognitive and psychosocial dimensions influencing healthy food choices among Moroccan consumers. Current scientific literature lacks comprehensive investigations into: (i) population-level understanding of nutritional principles, (ii) cultural attitudes toward healthy eating, and (iii) barriers to implementing dietary knowledge. These dimensions are crucial for designing effective, culturally appropriate interventions (HIND and BENMOUSSA Hind and Benmoussa, 2019). Yet, consumer perceptions of healthy eating remain largely underexplored in the Moroccan context (Salaheddine et al., 2015).

To fill this gap, this study employs a knowledge–Attitude–Practice (KAP) approach to examine Moroccan consumers' nutritional awareness, beliefs, and behaviors. Through a cross-sectional design, it offers empirical insights into the factors influencing healthy food choices. The findings aim to support the development of targeted health policies and educational programs aligned with Morocco's evolving food environment.

## 2 Materials and methods

### 2.1 Study design and area

We conducted a cross-sectional survey from October 2024 to January 2025 in the 12 regions of Morocco. The study aimed to evaluate knowledge about healthy food choices, attitudes regarding these choices, and actual practices related to healthy eating.

### 2.2 Ethical statement

All procedures involving human beings have been reviewed and approved by the Research Ethics Committee of Sultan Moulay Slimane University.

### 2.3 Study population and data collection

The target population for the study consisted of Moroccan individuals. A convenience sampling approach was employed, with participants recruited via WhatsApp, Facebook, Instagram and *E*-mail. Recruitment messages were disseminated through social media platform used in Morocco, providing detailed information about the study and including a link to a secure online questionnaire (Google forms). The survey was available in Arabic and French to accommodate the diverse linguistic population of Morocco. Income data were originally collected in Moroccan Dirhams (MAD) and converted to US Dollars (USD) using the average exchange rate of 10 MAD = 1 USD, based on central bank rates during the study period. A total of 1035 responses were collected, which was considered an adequate sample size for the scope of the study.

### 2.4 Sample size calculation

The sample size was determined using Cochran's formula: n₀ = Z^2^pq/e^2^, with Z = 1.96 (95 % confidence), e = 0.05, and *p* = 0.5 due to lack of prior data. The initial estimate was n₀ = 385. Using the adjusted Cochran's formula for a finite population (*N* = 36,828,330), the required minimum sample size remained 385.

### 2.5 Questionnaire design and outcome measures

The questionnaire was developed by adapting items from validated KAP surveys identified through a systematic literature review (A. Alissa, 2024; Fernandez-Alonso et al., 2024; Wertheim-Heck and Raneri, 2019), followed by refinement via structured focus group discussions with Moroccan experts for cultural appropriateness. The survey consisted of 38 questions grouped into four distinct sections: (a) sociodemographic characteristics, knowledge, attitudes and practices related to healthy food choices.

The sociodemographic characteristics section collected basic information such as gender, age, place of residence, level of education, and income.

The knowledge assessment section evaluated healthy food choices with three possible responses (“correct,” “incorrect,” “do not know”), using a scale adapted from previous validated studies (Osaili et al., 2017). In this section, each question answered “correct” was assigned one point, while “incorrect” or “ do not know” received zero points, except questions number one and two. For these two questions, one point was awarded for “incorrect” while zero points were assigned for “correct” or “don't know”. Participants' total score ranged from zero to seven.

The attitudes section utilized a scale with three response options (“agree”, “disagree”, and “do not know”), The scale was adapted from the study by Osaili et al. (2017). Each “agree” response received one point, while “disagree” or “do not know” responses were scored zero points. The total score for the attitudes section ranged from zero to eight.

The practices section was evaluated using a five-point ordinal scale (zero to four), with response options: Never = 0, Rarely = 1, Sometimes = 2, Often = 3, and Always = 4. However, for four specific questions (items 3, 4, 6, and 7), the scoring was reversed, where the responses designated as follows (Never = 4, Rarely = 3, Sometimes = 2, Often = 1, Always = 0). The total score for healthy food choices ranges from zero to 44.

The total scores of the participants range from zero to 59, with higher scores indicative of a higher level of knowledge, attitudes, and practices.

### 2.6 Validity and reliability of the questionnaire

To ensure validity, the survey was translated into Arabic, reviewed, and back-translated by independent bilingual researchers (Al Banna et al., 2022). Reliability was assessed through pilot testing (*n* = 20) to check clarity and internal consistency using Cronbach's alpha, which showed acceptable reliability for knowledge (α = 0.76), attitudes (α = 0.79), and practices (α = 0.71). Pilot data were excluded from analysis.

### 2.7 Statistical analyses

Multiple linear regression was used to identify factors associated with KAP scores, with categorical variables converted to dummies. Coefficients represent adjusted mean effects relative to reference categories. All variables were included, with *p* < 0.05 considered significant. Analyses were performed in SPSS v26.

## 3 Results

### 3.1 Sociodemographic characteristics

This study, which received 1035 valid responses from a total of 1042 questionnaires collected, revealed several key findings. The sample consisted of 52.3 % female participants. The majority of respondents were aged between 25 and 64 years (67.1 %). In terms of place of residence, urban participants accounted for 82.9 % of the total sample. Regarding the level of education, the data (Table 1) showed that 42.3 % of respondents had Bachelor's degree. Additionally, 553 participants (53.4 %) had a monthly income of less than 400 USD.

**Table 1:** Sociodemographic characteristics of Moroccan adults surveyed in 2024–2025.

Variables and Category	Frequency (n)	Percent (%)
Gender		
Female	541	52.3
Male	494	47.7
Age groups (years)		
18–24	334	32.3
25–64	695	67.1
>65	6	0.6
Place of residence		
Rural	177	17.1
Urban	858	82.9
The region of residence		
Béni Mellal-Khénifra	406	39.2
Casablanca-Settat	156	15.1
Dakhla-Oued Ed-Dahab	8	0.8
Drâa-Tafilalet	29	2.8
Fès-Meknès	60	5.8
Guelmim-Oued Noun	6	0.6
Laâyoune-Sakia El Hamra	20	1.9
Marrakech-Safi	138	13.3
Oriental	32	3.1
Rabat-Salé-Kénitra	82	7.9
Souss-Massa	59	5.7
Tanger-Tétouan-Al Hoceïma	39	3.8
Education level		
Primary	15	1.4
Secondary middle school	39	3.8
Secondary high school	155	15.0
Bachelor's degree	438	42.3
Master's degree or above	388	37.5
Monthly income groups USD		
<400	553	53.4
400–800	306	29.6
801–1200	93	9.0
1201-1600	51	4.9
>1600	32	3.1

### 3.2 Healthy food choices knowledge

The initial objective of this study was to assess participants' knowledge related to healthy food choices. This was measured using a knowledge scale comprising seven items. The average score obtained was 4.46 (SD = 1.2), which is significantly higher than the reference average score of 3.5, as confirmed by a one-sample *t*-test (*p* < 0.01). These findings suggest that participants had a relatively strong understanding of healthy eating principles.

Participants demonstrated a range of nutritional knowledge levels across the evaluated items. The highest proportion of correct responses was observed for the statement “Consuming fruits and vegetables is beneficial for health” (97.8 %). In contrast, lower correct response rates were noted for statements such as “White bread serves as a source of dietary fiber” (41.9 %) (Table 2).

**Table 2:** Knowledge and perceptions of healthy food choices among Moroccan adults, assessed via a seven-item questionnaire (2024–2025).

Item	Responses, n (%)		
	**Correct**	**Do not know**	**Incorrect**
1.Cholesterol is present solely in foods derived from animal sources.	280 (23)	272(26.3)	525(50.7)
2.White bread serves as a source of dietary fiber.	341(32.9)	260 (25.1)	434(41.9)
3.Fruits contain a higher amount of protein compared to meat.	158 (15.3)	159(15.4)	718(69.4)
4.Consuming foods and drinks that are high in calories is harmful to health.	703 (67.9)	141(13.6)	191(18.5)
5.Foods and drinks with high calorie content contribute to weight gain.	906 (87.5)	85 (8.2)	44 (4.3)
6.A person with high cholesterol should lower their intake of fatty foods.	881(85.1)	94(9.1)	60(5.8)
7.Consuming fruits and vegetables are beneficial for health.	1012(97.8)	7(0.7)	16(1,5)

### 3.3 Healthy food choices attitudes

The second research question explored participants' attitudes toward making healthy food choices. These attitudes were evaluated using an eight-item scale specifically designed to assess perceptions related to healthy eating. The average total score was 6.32 (SD = 1.23), indicating a generally favorable disposition toward healthy eating behaviors. A one-sample *t*-test confirmed that this mean score was significantly higher than the reference value of four (*p* < 0.01).

Overall, participants exhibited positive attitudes regarding healthy food consumption. A large proportion agreed with statements that promote health, such as the importance of regularly eating fruits and vegetables (96.4 %). Nevertheless, a certain level of ambiguity was noted; for instance, 32.7 % of respondents acknowledged difficulty in determining which foods are genuinely healthy (Table 3).

**Table 3:** Attitudes toward healthy eating among Moroccan adults, measured via an eight-item Likert scale (2024–2025).

Item	Responses, n (%)		
	Agree	Disagree	Do not know
1.Reducing the intake of salt and sugar in the diet can lead to improvements in overall health.	934(90.2)	16 (1.5)	85 (8.2)
2.Regular consumption of fruits and vegetables is a significant behavior that contributes to the promotion of health.	998 (96.4)	4 (0.4)	33 (3.2)
3.Excessive consumption of fat-rich foods is harmful to health.	901 (87.1)	23 (2.2)	111 (10.7)
4.There is a link between our diet and the diseases that affect us.	974 (94.1)	8 (0.8)	53 (5.1)
5.I am aware of how to select foods that benefit my health.	605 (58.5)	77(7.4)	353 (34.1)
6.The consumption of foods containin trans fats is harmful to health.	802 (77.5)	28 (2.7)	205 (19.8)
7.I believe it is hard to determine which foods are healthy.	338 (32.7)	378 (36,5)	319(30.8)
8.Excessive addition of sugar to juices and beverages is harmful to health.	990 (95.7)	17 (1.6)	28 (2.7)

### 3.4 Healthy food choices practices

The final research question focused on examining participants' dietary practices related to healthy food choices. These practices were evaluated using an 11-item scale specifically designed for this purpose. The overall mean score obtained was 25.83 (SD = 4.69), which is marginally higher than the established reference average of 22, suggesting a slightly above-average adherence to healthy eating behaviors.

The findings indicated the presence of a mixture of dietary habits among the respondents. While a high proportion reported healthy practices, such as frequent or consistent consumption of fresh fruits (89.7 %; 49 % often +40.7 % always), vegetables (89 %; 52.7 % often +36.3 % always), and fiber-rich foods (85.6 %; 45.3 % often +40.3 % always), other behaviors deviated from nutritional guidelines. For example, 21 % of participants (12.9 % often +8.1 % always) habitually added salt to meals after cooking, and only 40.5 % reported often or always checking food labels before purchase. Additionally, 34.2 % preferred ready-made meals over home cooking (Table 4).

**Table 4:** Self-reported dietary practices related to healthy food choices among Moroccan adults, assessed via an 11-item scale (2024–2025).

Item	Responses, n (%)				
	**Never**	**Rarely**	**Sometimes**	**Often**	**Always**
1. Do you consume fresh fruits such as apples, oranges, bananas, etc.?	2 (0.2)	45(4.3)	60(5.8)	507(49)	421(40.7)
2. Do you consume fresh vegetables such as carrots, lettuce, etc.?	6 (0.6)	53(5.1)	55(5.3)	545(52.7)	376 (36.3)
3. Do you drink sweetened beverages such as soda, sweetened fruit juices?	60 (5.8)	342 (33)	125 (12.1)	322 (31.1)	186(18)
4. Do you add salt to your food even after cooking?	230 (22.2)	285 (27.5)	302 (29.2)	134 (12.9)	84 (8.1)
5. Do you consume fiber-rich foods such as Whole grain bread, oats, etc.?	8(0.8)	83(8)	58 (5.6)	469 (45.3)	417 (40.3)
6. Do you consume fried foods such as fries, fried chicken, etc.?	33(3.2)	374 (36.1)	294(28.4)	305 (29.5)	29(2.8)
7. Do you consume foods high in fat such as fatty meats, cheese, oils, etc.?	10 (1)	86(8.3)	146 (14.1)	599 (57.9)	194 (18.7)
8. Do you try to reduce your sugar intake in drinks, sweets, etc.?	247(23.9)	331(32)	320 (30.9)	93(9)	44(4.3)
9. Do you buy products with low calorie or fat content?	73(7.1)	168(16.2)	430 (41.5)	294 (28.4)	70(6.8)
10. Do you make sure to read food labels before purchasing products?	62(6)	176(17)	378(36.5)	249(24.1)	170(16.4)
11. Do you prefer cooking meals at home rather than buying ready-made meals?	354(34.2)	18 (1.7)	170(16.4)	493(47.6)	0(0)

### 3.5 Associations between sociodemographic factors and knowledge, attitudes, and practices

Table 5 showed significant associations of several factors (demographic characteristics) with knowledge, attitudes, and practices scores about healthy food choices. The results indicated that there were no significant differences found between regions for knowledge, attitudes, or practices (all *p-*values >0.05). However, the correlation between residential location and the knowledge of healthy food choices was found to be statistically significant, with urban residents scoring higher than their rural counterparts (4.48 ± 1.16 vs. 4.37 ± 1.38; *p* = 0.01).

**Table 5:** Associations between sociodemographic factors and knowledge, attitudes, and practices scores related to healthy food choices in Moroccan adults (2024–2025).

Characteristics	Knowledge Score		Attitudes Score		Practices Score	
	Mean ± SD	*p-v*alue	Mean ± SD	*p-v*alue	Mean ± SD	*p-v*alue
Gender		<0.01		<0.01		0.32
Female	4.53 ± 1.08		6.27 ± 1.11		26.16 ± 4.79	
Male	4.4 ± 1.3		6.38 ± 1.35		25.47 ± 4.57	
Age groups (years)		<0.01		<0.01		<0.01
18–24	4.3 ± 1.23		6.05 ± 1.28		24.72 ± 4.44	
25–64	4.55 ± 1.17		6.45 ± 1.18		26.37 ± 4.7	
>65	3.67 ± 2.07		6.33 ± 1.86		25.17 ± 6.6	
Place of residence		<0.01		0.15		0.33
Rural	4.37 ± 1.38		6.3 ± 1.31		25.64 ± 4.5	
Urban	4.48 ± 1.16		6.33 ± 1.21		25.87 ± 4.73	
the region of residence		0.6		0.2		0.81
Beni Mellal-Khenifra	4.46 ± 1.15		6.36 ± 1.12		26.15 ± 4.65	
Casablanca-Settat	4.42 ± 1.04		6.21 ± 1.16		25.44 ± 4.7	
Dakhla-Oued Ed-Dahab	5 ± 0.75		6.63 ± 1.5		24.75 ± 2.19	
Draa-Tafilalet	4.55 ± 1.18		6.62 ± 1.4		25.97 ± 3.78	
Fes-Meknes	4.50 ± 1.22		6.28 ± 1.2		25.67 ± 3.66	
Guelmim-Oued Noun	4.00 ± 1.26		7.17 ± 0.75		24.83 ± 2.63	
Laayoune-Sakia El Hamr	4.10 ± 1.65		5.95 ± 1.61		26.00 ± 5.24	
Marrakech-Safi	4.53 ± 1.22		6.20 ± 1.26		25.62 ± 5.15	
Oriental	4.06 ± 1.4		6.16 ± 1.73		25.72 ± 4.31	
Rabat-Sale-Kenitra	4.52 ± 1.39		6.40 ± 1.32		26.26 ± 5.20	
Souss-Massa	4.56 ± 1.39		6.24 ± 1.45		25.63 ± 4.77	
Tanger-Tetouan-Al Hoceïma	4.49 ± 1.23		6.69 ± 1.13		24.74 ± 5.04	
Education level		0.03		0.02		0.03
Primary	4.07 ± 1.83		5.87 ± 2.23		23.73 ± 3.77	
Secondary middle school	4.64 ± 1.27		6.92 ± 1.26		25.67 ± 3.93	
Secondary high school	4.44 ± 1.28		6.36 ± 1.59		25.43 ± 4.46	
Bachelor's degree	4.35 ± 1.15		6.27 ± 1.13		25.57 ± 4.64	
Master's degree or above	4.59 ± 1.18		6.32 ± 1.1		26.38 ± 4.88	
Monthly income groups USD		0.06		0.02		<0.01
<400	4.39 ± 1.25		6.22 ± 1.33		25.3 ± 4.45	
400–800	4.54 ± 1.1		6.39 ± 1.09		25.97 ± 4.61	
801–1200	4.66 ± 1.21		6.59 ± 1.09		27.57 ± 5.07	
1201-1600	4.23 ± 1.26		6.33 ± 1.26		26.96 ± 5.22	
>1600	4.72 ± 1.09		6.69 ± 0.78		26.69 ± 5.80	

Note: *p*-values derived from independent samples t-test (gender and place of residence comparisons) and one-way ANOVA (other group comparisons). SD = standard deviation.

The relationship between perceptions about healthy food choices and the gender of the participants was examined by *t*-test. A significant difference was found in both the knowledge and attitude scores between females and males. The comparative analysis of the data revealed that female participants demonstrated higher levels of knowledge (4.53 ± 1.08 vs. 4.39 ± 1.32; *p* < 0.01), while male participants exhibited marginally higher attitudes scores (6.38 ± 1.35 vs. 6.27 ± 1.1; *p* *<* *0.01*). However, the study found no statistically significant differences in practices between the two groups.

One-way ANOVA statistical test was used to investigate the relationship between perceptions of healthy food choices and education level. Secondary middle school graduates were linked to better knowledge (4.64 ± 1.26) and attitudes (6.92 ± 1.27), while those with a master's degree or higher scored highest levels in practices (26.38 ± 4.89). The results indicated significant differences among age groups. Participants between the ages of 25 and 64 years exhibited the highest scores in knowledge (4.55 ± 1.17), attitudes (6.45 ± 1.18), and practices (26.37 ± 4.71). In contrast, participants over the age of 65 demonstrated the lowest mean scores in knowledge (3.67 ± 2.07; *p* = 0.01).

Furthermore, the monthly income was significantly related to both attitudes (*p* = 0.02) and practices (*p* < 0.01). The most favorable outcomes were observed in higher income brackets (>800USD), with the 801–1200 USD group demonstrating peak practice scores (27.57 ± 5.08), although knowledge did not show a significant difference (*p* = 0,06).

The results of the multiple regression analysis revealed statistically significant associations between certain sociodemographic characteristics and the levels of knowledge, attitudes, and practices related to healthy food choices (Table 6). For attitudes, the comparison revealed a higher proportion of positive attitudes among participants between the ages of 25 and 64 in comparison with those between the ages of 18 and 24 (*β* = 0.21, *p* = 0.02). The Guelmim-Oued Noun region displayed particularly favorable attitudes (*β* = 0.98, *p* = 0.04). Middle school (*β* = 1.05, *p* < 0.01) and high school education (*β* = 0.63, *p* = 0.05) were associated with better attitudes. Concerning practices, males scored significantly lower than females (*β* = −0.9, *p* = 0.02). The 25- to 64-year-old age group demonstrated better practices compared to the 18- to 24-year-old group (*β* = 0.97, *p* < 0.01). The Tanger-Tetouan-Al Hoceïma region demonstrated poorer practices (*β* = −1.63, *p* = 0.03).

**Table 6:** Multiple linear regression analysis of factors associated with healthy food choice knowledge, attitudes, and practices scores among Moroccan adults (2024–2025).

Characteristics	Model 1: Knowledge		Model 2: Attitudes		Model 3: Practices	
	*β*	95% CI	*Β*	95% CI	*β*	95% CI
Gender						
Female	1		1		1	
Male	-0.18	-0.28,0.22	0.08	-0.11,0.28	-0.89	-1.7, -0.12
Age groups (years)						
18-24	1		1		1	
25-64	0.046	-0.13, 0.22	0.21	0.38,2.31	0.97	0.28,1.67
>65	-0.79	-1.75, 0.16	0.53	1.50,1.08	1.38	-2.5,5.24
Place of residence						
Rural	1		1		1	
Urban	0.07	-0.12,0.26	0.006	-0.19,0.20	0.15	-0.62,0.92
The region of residence						
Beni Mellal-Khenifra	1		1		1	
Casablanca-Settat	0.05	-0.17,0.27	-0.07	-0.29, 0.15	-0.55	-1.43,0.32
Dakhla-Oued Ed-Dahab	0.6	-0.21, 1.39	0.05	-0.76, 0.86	-1.59	-4.8,1.62
Draa Tafilalet	0.03	-0.4,0.45	0.16	-0.27,0.59	-0.54	-2.3,1.18
Fes-Meknes	0.08	-0.27, 0.5	-0.12	-0.43, 0.19	-0.53	-1.8,0.7
Guelmim-Oued Noun	-0.6	-1.53,0.36	0.98	0.03,1.9	0.32	-3.5,4.1
Laayoune-Sakia El Hamra	-0.13	-0.65,0.38	-0.33	-0.85,0.19	0.40	-1. 7,2.48
Marrakech-Safi	0.14	-0.08,0.36	-0.13	-0.35,0.09	-0.64	-1.5,0.26
Oriental	-0.35	-0.75,0.06	-0.08	-0.49,0.33	0.25	-1.39,1.9
Rabat-Sale-Kenitra	0.02	-0.26,0.29	-0.05	-0.32,0.23	-0.14	-1.24,0.96
Souss-Massa	0.18	-0.13,0.49	-0.17	-0.48,0.15	-0.31	-1.6,0.94
Tanger-Tetouan-Al Hoceïma	-0.06	-0.43,0.32	0.36	-0.01,0.74	-1.63	-3.13, -0.12
Education level						
Primary	1		1		1	
Secondary middle school	0.04	-0.65, 0.74	1.05	0.35,1.75	0.92	-1.9,3.72
Secondary high school	0.06	-0.57, 0.69	0.63	-0.01,1.25	1.3	-1.24,3.84
Bachelor’s degree	-0.03	-0.64,0.59	0.59	-0.03,1.22	1.28	-1.21,3.77
Master’s degree or above	0.22	-0.41,0.84	0.45	-0.18,1.09	1.44	-1.08,3.96
Monthly income groups USD						
<400	1		1		1	
400–800	0.06	-0.12,0.23	0.02	-0.15,0.2	0.35	-0.35,1.05
801-1,200	-0.04	-0.32,0.23	0.17	-0.11,0.45	1.65	0.55,2.8
1,201-1,600	-0.35	-0.7, -0.01	0.08	-0.28,0.43	1.58	0.18,2.98
>1,600	-0.02	-0.45,0.40	0.32	-0.11,0.75	0.73	-0.99,2.44
Score						
Knowledge Score	--	--	0.32	0.26, 0.38	0.39	0.14,0.64
Attitudes Score	0.31	0.25,0.4	--	--	0.53	0.28,0.77
Practices Score	0.02	0.01,0.04	0.03	0.02,0.05	--	--

Note. CI = Confidence Interval; β = Regression coefficient; 1.00 = Reference category. The R² for adjusted model 1, 2 and 3 was 0.14, 0.16 and 0.09, respectively.

## 4 Discussion

This cross-sectional survey, based on the KAP model and conducted for the first time in Morocco, evaluates the population's knowledge, attitudes, and practices regarding healthy food choices and identifies sociodemographic factors influencing these dimensions. As unhealthy eating habits can adversely affect health, understanding KAP toward healthy food choices is essential. This study revealed three key findings: (1) urban residents showed significantly better nutrition knowledge compared to their rural counterparts (*p* < 0.01), (2) income level exhibited a strong correlation with healthy practices (*β* = 0.53), and (3) a notable gap existed between knowledge and actual dietary behaviors.

### 4.1 Knowledge about healthy food choice

The results of the present study indicate that the participants demonstrated a generally satisfactory level of knowledge regarding healthy eating (mean score: 4.46/7), which is consistent with the findings of previous research conducted in Malaysia and Saudi Arabia (A. Alissa, 2024; Yee, 2022). Participants demonstrated particularly high knowledge of the benefits of fruits and vegetables (97.8 % correct responses), which aligns with studies conducted in Kuwait (Kana”An et al., 2021) and in Ireland (Liu et al., 2020). These findings are consistent with the recommendations of the World Health Organization regarding the consumption of five essential food groups: grains, fruits and vegetables, dairy products, meats, and fish (WHO, 2020). However, a contradictory trend has been reported by other researchers in Saudi Arabia, who have indicated a poor knowledge regarding nutrition in that country (Sabur et al., 2022). Such discrepancies may stem from differences in sample demographics or educational backgrounds.

### 4.2 Attitudes toward healthy eating

Participants demonstrated positive attitudes toward healthy eating (mean score: 6.32/8), with strong awareness of the risks associated with excessive sugar consumption (95.7 % agreed). Contradictorily, a previous research indicated an evolution of sugar consumption among the Moroccan population (Allali, 2017). However, nearly one-third (32.7 %) struggled to accurately identify healthy foods, indicating persistent gaps in knowledge despite generally favorable attitudes. This contrasts with the findings of Mumena and colleagues in Saudi Arabia, reporting less favorable attitudes toward sugar intake (Mumena et al., 2020). The discrepancy may reflect cultural differences in dietary norms or variations in public health education initiatives.

### 4.3 Dietary practices

Regarding dietary practices, the respondents demonstrated moderate adherence to healthy eating behaviors (mean score: 25.83/44), while the frequent fruit (89.7 %) and vegetable (89 %) consumption aligned with Mediterranean dietary patterns (Bach-Faig et al., 2011). However, when focusing on the findings obtained in Kenitra City in Morocco, the frequencies of daily consumption of fruit (31.4 %) and vegetables (28 %) were relatively low. In addition, other practices revealed significant gaps. For instance, 34.2 % of the respondents reported a preference for ready-made meals, a figure that aligns with daily fast-food consumption rates in Settat City in Morocco (20.3 %) as documented by Talha et al. (2024), and reflects broader dietary shifts observed in low- and middle-income countries (Banna et al., 2022). Moreover, 21 % of the respondents habitually added salt after cooking, and only 40.5 % regularly checked food labels. These low engagement levels with nutritional information suggest a limited internalization of epistemic values—such as curiosity and awareness regarding food content—which are essential for informed dietary choices, as highlighted in the Pakistani context (Raza et al., 2021). This disparity between knowledge and behavior mirrors observations in Iran, where strong theoretical understanding often failed to translate into practices (Ahadi et al., 2014). These insights highlight the pressing need for targeted interventions, such as participatory cooking workshops or subsidized healthy meal initiatives, designed to bridge the gap between knowledge and practice. Such strategies are in line with the recommendations of El Rhazi et al. (2012) to promote adherence to the Mediterranean diet while addressing contemporary dietary challenges. This dual focus on cultural preservation and structural change is a distinctive feature of the proposed approach.

### 4.4 Sociodemographic influences on KAP

The results of this study highlight the complex influence of sociodemographic factors on knowledge, attitudes, and practices related to healthy food choices in Morocco. Urban residents demonstrated significantly higher nutritional knowledge than their rural counterparts (4.48 vs. 4.37; *p* < 0.01). Particularly, the study reflects regional trends observed in Kenitra and Settat, where disparities in access to nutritional education and diverse food options are evident (Hindi et al., 2024; Talha et al., 2024). This finding reinforces established urban-rural disparities in dietary behaviors and aligns with global trends (FAO, 2020).While women demonstrated greater nutritional knowledge (4.53 vs. 4.39; *p* < 0.01), men exhibited slightly more positive attitudes (6.38 vs. 6.27; *p* < 0.01), suggesting gendered roles in food management. Women, who are often responsible for household meal planning, have been shown to internalize dietary information more deeply. In contrast, men, influenced by social norms that favor convenience, were found to adopt optimistic attitudes without necessarily translating them into practices. This dichotomy is consistent with the findings of Raza et al. (2021) on emotional and conditional values.

Furthermore, the findings of the present study indicate a substantial correlation between educational attainment and KAP outcomes, particularly among secondary school graduates (knowledge: 4.64; attitudes: 6.92) and individuals with master's degrees (practices: 26.38). This observation underscores the pivotal role of advanced literacy in the application of knowledge, a phenomenon that has been documented in low- and middle-income countries, such as India (Sathiyamoorthi et al., 2025), Vietnam (Wertheim-Heck and Raneri, 2019), and Brazil (Gomes et al., 2019). Paradoxically, the highest practice scores among 25–64-year-olds (26.37) contrast with the challenges faced by seniors (>65 years: 3.67), reflecting both exposure to digital health campaigns targeting working-age adults and the gradual exclusion of older populations from modern information systems. These results, combined with significant KAP correlations (attitudes-practices: *β* = 0.53; *p* < 0.01), confirm the need for multidimensional interventions that balance cultural preservation (promoting the Mediterranean diet) with structural innovations (subsidies for rural households, labeling reforms) as previously recommended by El Rhazi et al. (2012),.

Higher-income groups (>800 USD) showed more favorable attitudes and practices (*p* < 0.05). This observation is consistent with the findings of previous studies that have established a correlation between economic constraints and the consumption of unhealthy foods (A. Alissa, 2024; Sabur et al., 2022). Notably, the Guelmim-Oued Noun region's elevated scores (*β* = 0.98, *p* = 0.04) may reflect local dietary traditions, meriting qualitative exploration. These findings align with those reported on Indonesia (Mailinda and Lestari, 2019) and Oman (Ali et al., 2020), where higher-income individuals exhibited better KAP.

### 4.5 Strengths and limitations

The present study represents the first national assessment of knowledge, attitudes, and practices related to food choices in Morocco using a culturally-adapted and validated approach. Its major strength lies in the use of robust multivariate analyses (including adjusted regressions) which enabled the identification of key sociodemographic determinants. However, several limitations should be acknowledged. First, the cross-sectional design does not allow conclusions on causality. Second, while our sample covered all the 12 Moroccan regions, the convenience sampling approach (via social media) may limit the representation of rural, elderly, and less digitally connected populations.

### 4.6 Practical implications

This study highlights the need for context-specific strategies to improve dietary practices in Morocco. While knowledge and attitudes toward healthy eating are moderately positive, practical application is lacking, especially in rural and low-income groups. Priorities include targeted education on label use and meal planning, simplified front-of-pack labeling, better nutrition communication, integration of nutritionists into primary care, and community-based programs to address malnutrition and promote sustainable change.

## 5 Conclusion

This study reveals a persistent paradox in Moroccan nutrition patterns: while theoretical knowledge and attitudes about healthy eating are generally strong, practical implementation remains inconsistent, with significant disparities across urban/rural, socioeconomic, and age

groups. The findings highlight three critical pathways for improvement: (1) structural efforts to improve access—via school nutrition programs, rural cooking workshops, and subsidies for low-income groups. (2) targeted education to correct misconceptions (i.e. white bread, protein sources) while strengthening practical skills such as label reading and home meal preparation. (3) further research to understand sociocultural and economic barriers, especially in specific regions such as Guelmim-Oued Noun. By addressing immediate gaps through targeted programs and building evidence for long-term solutions, Morocco has the potential to transform nutritional knowledge into sustainable eating practices. This integrated approach is a model for similar contexts where traditional dietary knowledge coexists with modern lifestyle challenges.

## CRediT authorship contribution statement

**Morad Kaddouri:** Writing – original draft, Methodology, Investigation, Formal analysis, Data curation. **Omar Ait El Alia:** Writing – original draft, Methodology, Investigation. **Mohamed Bouhrim:** Writing – review & editing, Supervision, Data curation, Conceptualization. **Fahd A. Nasr:** Writing – review & editing, Resources, Project administration, Funding acquisition. **Ashraf Ahmed Qurtam:** Writing – review & editing, Project administration, Funding acquisition. **Said Ihbour:** Software, Formal analysis, Data curation. **Khalid Boutoial:** Writing – review & editing, Supervision, Investigation, Conceptualization.

## Funding

This work was supported and funded by the Deanship of Scientific Research at *Imam* Mohammad Ibn Saud Islamic University (IMSIU) (grant number IMSIU-DDRSP2501).

## Declaration of competing interest

The authors declare that they have no known competing financial interests or personal relationships that could have appeared to influence the work reported in this paper.

## Data Availability

Data will be made available on request.
